# To the Editors of the Medical and Physical Journal

**Published:** 1808-12

**Authors:** 


### *To the Editors of the Medical and Physical Journal.*

GENTLEMEN,

IF I had known that you admitted random *Hypercritics* into your Journal, I could have enabled you to spoil as much paper as you could wish. I allude to an objection in your last, to the term *Morbid Anatomy*. *Morbidus* is regularly derived from *morbus*, and can only mean, as all our dictionary writers translate these derivatives, "*of or belonging to*." The Latin is allowed to have a peculiar advantage above most other languages in its abundance of these adjectives, by which their writers escape the perpetual repetition of the genitive case. It is therefore less to be wondered, if your correspondent could find no appropriate word by which he could translate the term into any foreign tongue. This was, I believe, with Mr. Addison, a test of wit, but wit is seldom admitted into the title page of philosophical works. Even if it were, would it be fair to translate an expression backward and forward into the same language? Morbid is either English or Latin. If the first, why is it translated *diseased?* If the last, is it not a little *sickening* to see it translated *ægrotans?*

Dr. Soemmering has found no difficulty in procuring a

German word for *morbid*; and I cannot help remarking, that *Zergliederungskunst* is a somewhat unfair translation of *Anatomy*. The former, by its termination, must be confined to the *art* of anatomy; the latter may very well be translated *Bau*, as it is often used for *structure* by every medical writer. The *anatomy* of the human body; anatomy of the viscera, &c. The transition to *diseased anatomy* is very easy, when the diseased structure of individual parts is to be described.

Having mentioned Mr. Addison, permit me to produce an authority from the Golden Age of our language.

"*Glad chains.*] The ignorance of these moderns! 'Twas altered in one edition to Gold chains, shewing more regard to the metal of which the chains of Aldermen are made, than to the beauty of the Latinism or Grecism, nay of figurative speech itself. *Lætas segetes*, glad for making glad, &c.

SCRIBLERUS."

